# Colorectal carcinomas with microsatellite instability display a different pattern of target gene mutations according to large bowel site of origin

**DOI:** 10.1186/1471-2407-10-587

**Published:** 2010-10-27

**Authors:** Manuela Pinheiro, Terje Ahlquist, Stine A Danielsen, Guro E Lind, Isabel Veiga, Carla Pinto, Vera Costa, Luís Afonso, Olga Sousa, Maria Fragoso, Lúcio Santos, Rui Henrique, Paula Lopes, Carlos Lopes, Ragnhild A Lothe, Manuel R Teixeira

**Affiliations:** 1 Department of Genetics, Portuguese Oncology Institute - Porto, Rua Dr. António Bernardino Almeida, 4200-072 Porto, Portugal; 2 Department of Cancer Prevention, Institute for Cancer Research, The Norwegian Radium Hospital, Oslo University Hospital, Montebello, 0310 Oslo, Norway; 3 Centre for Cancer Biomedicine, University of Oslo, Montebello, 0310 Oslo, Norway; 4 Department of Pathology, Portuguese Oncology Institute - Porto, Rua Dr. António Bernardino Almeida, 4200-072 Porto, Portugal; 5 Department of Radiotherapy, Portuguese Oncology Institute - Porto, Rua Dr. António Bernardino Almeida, 4200-072 Porto, Portugal; 6 Department of Oncology, Portuguese Oncology Institute - Porto, Rua Dr. António Bernardino Almeida, 4200-072 Porto, Portugal; 7 Department of Surgery, Portuguese Oncology Institute - Porto, Rua Dr. António Bernardino Almeida, 4200-072 Porto, Portugal; 8 Faculty of Medicine, University of Oslo, Oslo, Norway; 9 Institute of Biomedical Sciences Abel Salazar (ICBAS), University of Porto, Largo Prof. Abel Salazar, 4099-003 Porto, Portugal

## Abstract

**Background:**

Only a few studies have addressed the molecular pathways specifically involved in carcinogenesis of the distal colon and rectum. We aimed to identify potential differences among genetic alterations in distal colon and rectal carcinomas as compared to cancers arising elsewhere in the large bowel.

**Methods:**

Constitutional and tumor DNA from a test series of 37 patients with rectal and 25 patients with sigmoid carcinomas, previously analyzed for microsatellite instability (MSI), was studied for *BAX*, *IGF2R*, *TGFBR2*, *MSH3*, and *MSH6 *microsatellite sequence alterations, *BRAF *and *KRAS *mutations, and *MLH1 *promoter methylation. The findings were then compared with those of an independent validation series consisting of 36 MSI-H carcinomas with origin from each of the large bowel regions. Immunohistochemical and germline mutation analyses of the mismatch repair system were performed when appropriate.

**Results:**

In the test series, *IGFR2 *and *BAX *mutations were present in one and two out of the six distal MSI-H carcinomas, respectively, and no mutations were detected in *TGFBR2*, *MSH3*, and *MSH6*. We confirmed these findings in the validation series, with *TGFBR2 *and *MSH3 *microsatellite mutations occurring less frequently in MSI-H rectal and sigmoid carcinomas than in MSI-H colon carcinomas elsewhere (*P *= 0.00005 and *P *= 0.0000005, respectively, when considering all MSI-carcinomas of both series). No *MLH1 *promoter methylation was observed in the MSI-H rectal and sigmoid carcinomas of both series, as compared to 53% found in MSI-H carcinomas from other locations (*P *= 0.004). *KRAS *and *BRAF *mutational frequencies were 19% and 43% in proximal carcinomas and 25% and 17% in rectal/sigmoid carcinomas, respectively.

**Conclusion:**

The mechanism and the pattern of genetic changes driving MSI-H carcinogenesis in distal colon and rectum appears to differ from that occurring elsewhere in the colon and further investigation is warranted both in patients with sporadic or hereditary disease.

## Background

Colorectal cancer is the third most common neoplasia in the Western world, preceded only by lung cancer in male and breast cancer in female [1]. Approximately 25 to 35% of colorectal cancers are located in the rectum. Multiple differences between cancer of the right and left colon and rectum with regard to epidemiological, clinical behavior, pathological and molecular features suggest that the mechanisms of sporadic colorectal carcinogenesis may differ according to tumor location [2,3]. A possible explanation for this could be the different embryological origin of the large intestine, as the ascending and two thirds of the transverse colon originate from the midgut and the last third of transverse, descending colon and rectum from the hindgut [4]. Although few studies have separately analyzed rectal cancer they indicate that approximately 80 to 98% of these cancers arise through the chromosomal instability pathway (CIN), presenting high mutational frequency in *APC*, *TP53*, and *KRAS *in addition to numerous chromosome changes [2,3,5]. Nonetheless, a few rectal cancers can develop through the microsatellite instability (MSI) pathway [2,5].

MSI is a widespread instability in coding and noncoding microsatellite sequences, due to mismatch repair (MMR) deficiency [6]. Through the MSI pathway, colorectal cancer progression is accelerated through accumulation of mutations in coding repetitive sequences of target genes with growth-related functions. However, most of the microsatellite mutations observed in MMR-deficient cells are bystander events that do not play a causal role in carcinogenesis. Criteria to clarify what constitutes a true MSI target gene have been proposed, although most studies have relied on mutation frequency data and on functional studies [7,8]. In colorectal cancer, mutations have been found in a number of genes with key cellular roles, such as growth factor receptors (*TGFBR2 *and *IGF2R)*, genes involved in apoptosis (*BAX*), as well as genes relevant for DNA repair (*MSH3*, *MSH6*) [6,9]. MSI frequency reported in rectal cancer is < 10%, but it is unknown whether or not the same target genes are involved [10].

*KRAS *and *BRAF *gene mutations are associated with colorectal development through both CIN and MSI pathways. These genes are members of the mitogen activated protein kinase (MAPK) pathway, which regulates cell proliferation, differentiation, senescence and apoptosis [11]. *KRAS *mutations are present in 30 to 50% of colorectal carcinomas, occurring mainly in codons 12 and 13 [12-14]. *BRAF *mutations are present in 3.7 to 21% of colorectal carcinomas, mainly in codon 600 [13,15-17]. Only a small number of studies have specifically addressed the mutational frequency of *KRAS *and *BRAF *in rectal carcinomas, reporting a mutational frequency of 21 to 46% for *KRAS *and about 4% for *BRAF *[12,13].

We have previously reported the frequency of nuclear (MSI) and mitochondrial instability in a series of rectal and sigmoid carcinomas [18]. In this study, we aimed to further contribute to the understanding of the pathogenetic mechanisms operating in distal colon and rectal cancers compared to those arising elsewhere in the large bowel.

## Methods

### Patient characteristics and DNA extraction

Our test series consisted of 37 rectal and 25 sigmoid cancer patients treated by surgical resection at the Portuguese Oncology Institute-Porto, which have previously been analyzed for nuclear (MSI) and mitochondrial instability (one rectal cancer was excluded from the initial series because the patient had received neoadjuvant treatment) [18]. As described, all tumor samples were paraffin embedded and reviewed by a pathologist (LA) and peripheral blood or normal mucosa was also collected from the same patients. The minimal percentage of tumor cells in the tissue sections was 50%. Clinical data were obtained from hospital records and tumor staging was performed using the American Joint Committee on Cancer (AJCC) criteria. Family history was assessed from hospital records and none of the patients presented a family or personal history indicative of familial adenomatous polyposis (FAP), MYH-associated polyposis (MAP) or hereditary non-polyposis colorectal cancer (HNPCC). This study was approved by the Institutional Review Board of the Portuguese Oncology Institute-Porto. DNA was isolated from paraffin-embedded tumor and normal mucosa as described by Lungu *et al *[19] and from peripheral blood using the salt-chloroform extraction method [20].

The data from an independent series of 36 MSI-H carcinomas was included for validation. These carcinomas were fresh frozen from patients treated in Norway and included eight caecum, eight ascending colon, six right flexure colon, four left colon (including three flexure/transverse and one descending colon), three sigmoid and seven rectal carcinomas [21]. This validation series included only sporadic tumors as determined by written questionnaires [21].

### Microsatellite instability and target gene analyses

MSI evaluation of the test series has been previously published by our group and was performed using the Bethesda panel of markers (BAT25, BAT26, D2S123, D5S346 and D17S250) and the 1997 National Cancer Institute guidelines [18]. MSI evaluation of the validation series was performed as for the test series [21].

Microsatellite sequences of the potential target genes *TGFBR2 *(A10)*, BAX *(G8)*, IGF2R *(G8)*, MSH3 *(A8) and *MSH6 *(C8) were analyzed by PCR and fragment analysis. PCR was carried out as previously described using fluorescence-labeled primers [22,23]. Fragments were analyzed for length variations on an ABI Prism 310 DNA sequencer (the test series) (Applied Biosystems, Foster City, CA, USA) and a 3730 DNA Analyzer (the validation series) (Applied Biosystems) and allele sizes were determined using Genemapper software (version 3.7, Applied Biosystems). The results were independently scored by two observers and a second round of analyses confirmed the results.

### *BRAF *exon 15 and *KRAS *exon 2 mutation screening

*BRAF *exon 15 and *KRAS *exon 2 (coding exon 1) were analyzed for mutations by direct sequencing on an ABI PRISM 310 automatic sequencer using Big Dye Terminator V1.1 Chemistry (Applied Biosystems), according to the manufacturer' s recommendations and as previously described [15]. Data analysis was performed by Sequencing Analysis software (version 5.2, Applied Biosystems). In the validation MSI-H carcinomas series, *BRAF *exon 15 and *KRAS *exon 2 mutation analyses had previously been performed in 27 and 36 of the 36 cases, respectively, as described by Ahlquist *et al *[24].

### *MLH1 *gene promoter methylation analysis

The methylation status of the *MLH1 *gene promoter was determined in the six MSI-H carcinomas from the test series and in the respective normal samples by two different techniques: methylation-specific multiplex ligation-dependent probe amplification (MS-MLPA) and methylation-specific PCR (MSP). MS-MLPA was performed according to the SALSA MS-MLPA ME001B Tumor suppressor-1 Kit (MRC-Holland) instructions. In order to confirm the results obtained by MS-MLPA, we performed MSP after chemical treatment of two μg of genomic DNA with sodium bisulfite, as previously described [25]. One set of methylation-dependent and unmethylation-dependent primers for the *MLH1 *gene promoter region covering the 1686-L1266 probe region of the TS1 MS-MLPA kit were designed using MethylExpress software (1.0 version, Applied Biosystems). Primer sequences for the unmethylated reaction were 5' -GGTTTTTTTGGTGTTAAAATGTT-3' (forward) and 5' -CTTAAATAAACCCAACTCAACTC-3' (reverse) and for the methylated reaction were 5' -TTTTTTGGCGTTAAAATGTC-3 (forward) and 5' - AAATAAACCCGACTCGACTC-3' (reverse).

In the validation MSI-H carcinomas series, *MLH1 *promoter methylation was analyzed in 25 of the 36 cases (three caecum, eight ascending colon, five right flexure, three left colon, two sigmoid and four rectal carcinomas). *MLH1 *promoter methylation of this series has previously been performed by MSP as described by Lind *et al *(2004) [26].

### MMR immunohistochemical analysis

Assessment of MLH1, MSH2, and MSH6 immunoexpression was evaluated in the six MSI-H carcinomas of the test series, with PMS2 being evaluated in the four MSI-H carcinomas with normal expression of the other three MMR proteins. Four μm sections were cut and placed in silanyzed slides. Immunostaining was performed using an avidin-biotin complex peroxidase method (Elite PK-6200, Vector, Burlingame, CA, USA). Briefly, after dewaxing the sections, endogenous peroxidase activity was inhibited with freshly prepared 0.5% hydrogen peroxide in distilled water for 20 min. Antigen retrieval was performed with EDTA buffer, pH8, for 40 minutes. Incubation with primary antibodies for MLH1 (Clone G168-15, BD Pharmingem, San Jose, CA, USA), MSH2 (Clone G219-1129, BD Pharmingem), MSH6 (Clone 44, BD Pharmingem) and PMS2 (Clone A16-4, Zytomed Systems, Berlin, Germany) was performed overnight at 4°C, at dilutions 1:100, 1:300, 1:1000, and 1:50 respectively, in 1% BSA in phosphate buffer saline (PBS). All incubations were performed in a humified chamber. Sections were developed with a peroxidase substrate solution (0.05% 3,3-diaminobenzidine tetrahydrocloride, 0.01% H_2_O_2 _in PBS), counterstained with hematoxylin, dehydrated and mounted. Appropriate positive and negative controls were used for each antibody, i.e., internal controls (non-tumor tissue) and external controls (cases with germline mutations at *MLH1*, *MSH2 *and *MSH6 *genes). Assessment of MLH1, MSH2, MSH6 and PMS2 immunoexpression was performed by light microscopy at x400 magnification by a pathologist (RH).

### Screening for *MSH2 *and *MSH6 *germline alterations

Genomic DNA from two rectal cancer patients with absent MSH2/MSH6 immunoreaction was screened for *MSH2 *and *MSH6 *germline mutations. *MSH2 *and *MSH6 *coding exons (except *MSH2 *exons one and five and *MSH6 *exon one and the acceptor splice site of exon 10) were studied by Denaturing Gradient Gel Electrophoresis (DGGE) using primers and conditions as described by Wu *et al *[27] and Ingeny (The Netherlands). Fragments with abnormal DGGE patterns and *MSH2 *exons one and five and *MSH6 *exon one and the acceptor splice site of exon 10 were analyzed by direct sequencing in an ABI PRISM 310 automatic sequencer using Big Dye Terminator Chemistry (Applied Biosystems), according to the manufacturer' s recommendations. Whenever necessary, *MSH6 *exon seven was re-sequenced using different set of primers to exclude or confirm the presence of a polymorphism at the initial primer annealing site [28].

*MSH2 *and *MSH6 *exonic rearrangements were screened by multiplex ligation-dependent probe amplification (MLPA), according to the SALSA MLPA P003 MLH1/MSH2 Kit and P072MSH6 (MRC-Holland, Amsterdam) instructions.

### Statistical analysis

Statistical analysis was carried out with SPSS version 15. Results were expressed in absolute frequencies and percentages. The statistical significance of no association between different variables was performed with the Fisher' s exact test. *P *values inferior to 0.05 were considered statistically significant.

## Results

### MSI target gene mutations and *MLH1 *methylation

**Figure 1:**
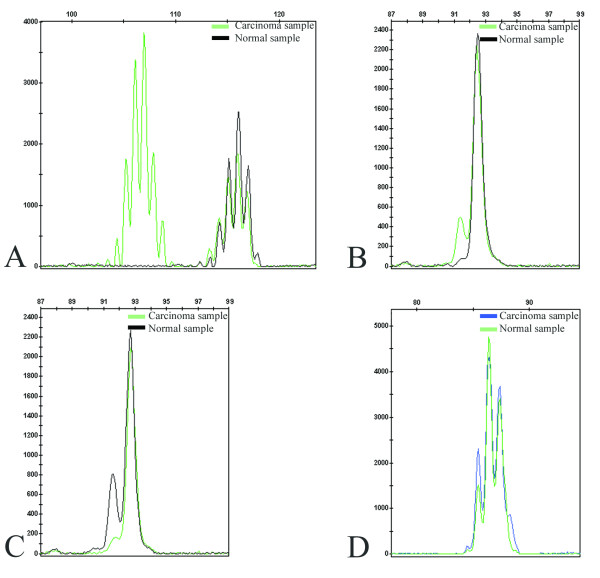
**Fragment analysis electrophorograms showing microsatellite sequences of BAT26 (A), *IGF2R *(G8) (B), *BAX *(G8) (C), and *TGFBR2 *(A10) (D) in a rectal carcinoma (T41)**. Microsatellite instability was found in BAT26, *IGF2R*, *BAX*, but not in *TGFBR2*.

Three of the 37 rectal and three of the 25 sigmoid carcinomas of the test series showed MSI-H [18]. Mutations in the MSI target genes *IGF2R *and *BAX *were found in one (33.3%) and two (66.7%) out of the three rectal MSI-H carcinomas, respectively (Figure 1; Table 1), whereas no mutations were detected in *TGFBR2*, *MSH3*, and *MSH6 *microsatellite sequences in this test series. None of the three MSI-H sigmoid carcinomas presented mutations in any of the target genes analyzed. Furthermore, none of the six MSI-H rectal and sigmoid carcinomas presented *MLH1 *gene promoter methylation. The histopathological features that characterize the MSI-H tumors of the test series are presented in Table 1. No association was found between the presence of MSI-H and clinicopathological features.

**Table 1:** Histopathologic features, *BAX *and *IGF2R *microsatellite mutation status, *BRAF *mutation status, MMR protein expression and mutation status in the rectal and sigmoid MSI-H carcinomas from the test series

Tumor location (sample ID)	Morphology	Differentiation	Growth pattern	Lymphocytic infiltration	Mucinous	*BAX*	*IGF2R*	*KRAS*	*BRAF*	MMR protein expression	*MMR *germline mutation
Rectum (T8)	Exophytic	Moderate/well	Expanding	Moderate	No	Positive	Negative	Negative	Negative	MSH2/MSH6 absence	*MSH2 *c.388_389del
Rectum (T20)	Ulcerated	Moderate/well	Expanding	Moderate	No	Negative	Negative	Negative	Negative	Normal	N.a.
Rectum (T41)	Ulcerated/exophytic	Moderate/well	Expanding	Moderate	Yes	Positive	Positive	Negative	Negative	MSH2/MSH6 absence	Negative
Sigmoid (T27)	Ulcerated	Well	Infiltrating	Sparse	No	Negative	Negative	Negative	Negative	Normal	N.a.
Sigmoid (T33)	Ulcerated/exophytic	Moderate/well	Expanding/infiltrating	Sparse	No	Negative	Negative	Positive	Negative	Normal	N.a.
Sigmoid (T35)	Exophytic	Poor	Infiltrating	Sparse	No	Negative	Negative	Negative	Positive	Normal	N.a.

MMR - Mismatch repair. MSI-H - microsatellite instability-high. T- test series; N.a. - not analyzed; *TGFBR2*, *MSH3 *and *MSH6 *mutations and *MLH1 *methylation were not found.

Intrigued by the low rate of target gene mutations and absence of *MLH1 *hypermethylation in MSI-H rectal and sigmoid carcinomas in the test series, we validated these findings in an independent series of MSI-H carcinomas arising from several locations of the large bowel (Additional file 1, Table S1). After grouping the two datasets we observed that the mutation frequency of the target genes was associated with large bowel carcinoma location (Table 2). *TGFBR2 *and *MSH3 *mutations were detected more frequently in right MSI-H colon carcinomas (*P *= 0.00005 and *P *= 0.0000005, respectively) than in MSI-H rectal and sigmoid carcinomas. *IGF2R*, *BAX *and *MSH6 *mutations were also detected more frequently in proximal colon carcinomas, but the difference was not statistically significant (Table 2). Furthermore, no *MLH1 *gene promoter hypermethylation was found in any of the MSI-H rectal or sigmoid carcinomas of the combined series, whereas it was found in 53% of carcinomas located elsewhere (*P *= 0.004).

**Table 2:** *TGFBR2*, *BAX*, *IGF2R*, *MSH3*, *MSH6 *microsatellite sequences, *BRAF *and *KRAS *mutation frequency and *MLH1 *promoter hypermethylation status in the MSI-H colorectal cancer according to the large bowel of origin (rectal/sigmoid carcinomas compared with those located elsewhere in the colon from both series)

Tumor location	*TGFBR2* (%)	*BAX* (%)	*IGF2R* (%)	*MSH3* (%)	*MSH6* (%)	*MLH1 *methylation (%)	*KRAS* (%)	*BRAF* (%)
Proximal Colon	23/26 (88)	12/26 (46)	8/26 (31)	20/26 (77)	5/26 (19)	10/19 (53)	5/26 (19)	9/21 (43)
Rectum/Sigmoid	4/16 (25)	3/16 (19)	3/16 (19)	0/16 (0)	1/16 (6)	0/12 (0)	4/16 (25)	2/12 (17)
	*P *= 0.00005	*P *= 0.102	*P *= 0.485	*P *= 0.0000005	*P *= 0.380	*P *= 0.004	*P *= 0.711	*P *= 0.249

MSI-H - microsatellite instability-high

### *KRAS *and *BRAF *mutations

**Figure 2:**
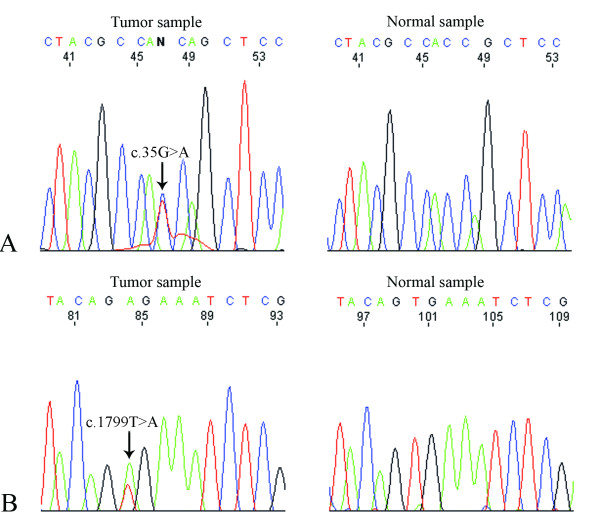
**Sequence electrophorograms showing *KRAS *and *BRAF *mutations**. A- Rectal carcinoma presenting the mutation c.35G > A (arrow) in *KRAS *exon 2 to the left and the normal tissue sample to the right (sequencing analysis is in the reverse direction); B- Sigmoid carcinoma presenting the mutation c.1799T > A (V600E) (arrow) in *BRAF *exon 15 to the left and the corresponding normal tissue sample to the right.

*KRAS *exon 2 mutations were detected in 13 (35.1%) of the 37 rectal carcinomas of the test series (Additional file 2, Table S2). All mutations occurred in codons 12 (69.2%) and 13 (30.8%) and the most frequent was c.35G > A (Figure 2A). None of the cases with *KRAS *mutations presented MSI-H, but two cases (15.4%) presented MSI-L. Five (20%) sigmoid carcinomas presented mutations in *KRAS *exon 2, all in codons 12 (80%) and 13 (20%), and the most frequent was also c.35G > A (Additional file 2, Table S2). One (20%) sigmoid carcinoma with *KRAS *mutation presented MSI-H. No mutations were detected in *BRAF *exon 15 in rectal carcinomas of the test series, but one (4%) MSI-H sigmoid carcinoma with no *KRAS *mutation presented the mutation c.1799T > A (V600E) (Figure 2B).

When considering the MSI-H carcinomas of the combined series, *KRAS *exon 2 mutations were detected in five (19%) colon carcinomas located proximally to the sigmoid and in four (25%) rectal/sigmoid carcinomas. *BRAF *exon 15 mutations were detected in nine (43%) proximal colon carcinomas and in two (17%) rectal/sigmoid carcinomas. No statistically significant association was found with tumor location (Table 2).

### MMR immunohistochemistry and *MSH2 */*MSH6 *germline mutations

Of the six MSI-H carcinomas from the test series, none of them presented criteria for MMR germline mutation analysis, namely family history or early onset of cancer (the average age of the patients with MSI-H carcinomas was 68 years). However, since none of them presented *MLH1 *promoter hypermethylation, MMR immunohistochemical analysis was performed and two rectal carcinomas showed absence of MSH2/MSH6 expression (Table 1). One of these patients was shown to present a pathogenic germline mutation in *MSH2 *exon 3 (Table 1). MLH1 expression was normal in all MSI-H carcinomas and PMS2 expression was normal in the four cases with normal MLH1, MSH2 and MSH6 expression. As far as can be ascertained from the information assessed from written questionnaires, the patients of the validation series did not fulfill the criteria for germline MMR mutation analysis and therefore the MSI-H carcinomas were considered sporadic [21].

## Discussion

Existing data on MSI-H frequency indicate that it varies from 10 to 20% in sporadic colorectal cancer, but varies from less than 10% in sporadic rectal carcinomas to about 40% in carcinomas from the right-sided colon [10,17,18,29]. About 70% of MSI-H sporadic colorectal cancers present *MLH1 *promoter hypermethylation [30]. In this study the *MLH1 *promoter was analyzed in regions D (in the test series) and C (in the MSI-H validation series), which are both strongly associated with MLH1 protein expression [31]. None of the rectal and sigmoid MSI-H carcinomas of both series presented *MLH1 *promoter hypermethylation, which we confirmed is significantly associated with colon tumors located more proximally (*P *= 0.004). These data are compatible with the recent observation of Watanabe *et al *[32] that *MLH1 *promoter methylation is significantly less common in left than in right MSI-H colorectal cancer. This difference is reflected in distinct gene expression profiles, which could be taken to indicate that left MSI-H colorectal cancer is a pathogenetically different subgroup among MSI-H sporadic carcinomas [32].

Genes with repetitive sequences located in coding regions are prone to mutations in colorectal carcinomas with the MSI-H phenotype. In fact, there is a well established association between an ineffective MMR system and mutations in the target genes that we have studied, which presumably play a relevant role in colorectal carcinogenesis through the MSI pathway [9,33]. In rectal MSI-H carcinomas of the test series, we detected mutations only in *IGF2R *and *BAX *genes, possible indicating that they are also target genes in the MSI pathway in rectal cancer. The *TGFBR2 *coding (A)10 sequence is among the most frequently mutated (70 to 90%) sites in MSI-H colorectal carcinomas, indicating that alterations in this gene are crucial for the development of MSI neoplasias [9,34]. Furthermore, the mutational frequency in MSI-H colorectal cancer is 20 to 39% for *MSH3 *and 30 to 40% for *MSH6 *[33,35]. Interestingly, we did not detect any mutation in *TGFBR2*, *MSH3*, or *MSH6 *microsatellite sequences in rectal or sigmoid MSI-H carcinomas in our test series. In order to confirm these findings, we compared our data with those of an independent series of MSI-H carcinomas with origin in each of the large bowel regions. The trend observed in the test series was confirmed in the validation set, and when all MSI-H carcinomas of the two series are grouped together we observed that *TGFBR2 *and *MSH3 *mutations were significantly more prevalent in proximal than in distal (sigmoid and rectal) cancers (*P *= 0.00005 and *P *= 0.0000005, respectively). This disparity suggests that these genes are not commonly involved in the development of rectal and sigmoid cancer through the MSI pathway or that alternative mechanisms of inactivation exist. Qualitative (type of target gene) and quantitative (number and frequency of altered target genes) differences have been observed regarding MSI-H target genes in different types of cancers. For instance, *TGFBR2 *mutational frequency is higher in MSI-H colon carcinomas (70 to 90%) than in MSI-H endometrium carcinoma (17 to 19%), suggesting that biological features and functional roles of target genes may differ depending on the tissue of tumor origin [33,34]. Our data suggest that both the mechanism of MSI-H and its target genes differ in colorectal carcinomas depending on large bowel site of origin.

Based on clinical data, germline mutations in the mismatch repair genes were initially considered unlikely. However, since none of the MSI-H rectal and sigmoid carcinomas presented *MLH1 *promoter methylation, we performed immunohistochemical staining to determine the expression of the MMR proteins MLH1, MSH2, MSH6 and PMS2. Two MSI-H rectal carcinomas showed absence of MSH2/MSH6 proteins and subsequent mutation analysis demonstrated that one of the patients presented a germline mutation in *MSH2 *exon 3. We did not detect any mutation either in the *MSH2 *or *MSH6 *genes in the second patient, although it may exist in the promoter or intronic regions not probed in this investigation. These two cases presented also alterations in the *BAX *and *IGF2R *genes in their carcinomas (Table 1), which are compatible with a constitutional MMR deficiency that leads to an acquired genetic instability. The immunohistochemical expression of all four MMR proteins in the remaining four cases does not necessarily imply normal DNA mismatch repair function, as missense mutations in *MSH2*, *MLH1 *or *MSH6 *genes may give rise to normal protein levels but abnormal function [36]. Although we did not perform MMR mutation analysis in the validation series, the patient age composition as a whole does not fit a Lynch syndrome profile, as the mean age in the validation series is 68 years, with only three cases unmethylated at the *MLH1 *promoter being of young age (33-41 years old), all rectal or sigmoid. Additionally, *de novo *germline mutations in other MMR components cannot be ruled out for neither the test (one rectal and the three sigmoid MSI-H tumors with normal MLH1, MSH2, MSH6, and PMS2 immunohistochemical staining) nor the validation series. As mentioned above, *TGFBR2*, *MSH3 *and *MSH6 *microsatellite sequences present high mutational rates in right MSI-H colorectal cancer, demonstrating that alterations in these genes are important for the development of MSI neoplasias [9,34]. The fact that we detected significantly less mutations in these three genes in distal MSI-H carcinomas, even in a rectal carcinoma arising in an individual with a MMR constitutional deficiency, could indicate that these genes are not essential for the cancer development in the sigmoid and rectum. It would be interesting to examine for location differences of other genes showing a high mutation frequency in MSI-H tumors such as *AC1*, *ACVR2A*, *HT001*, *MRE11A*, *PTHLH*, and *TAF1B*, which all carry mononucleotide repeats in the coding region and show a mutation frequency of ~70% or higher. However, the relevance of these genes in rectal tumorigenesis remains to be clarified [35].

Several studies have demonstrated the relevance of the MAPK signaling pathway in colorectal cancer, particularly involving alterations in the proto-oncogenes *KRAS *and *BRAF*. Deregulation of this pathway can result in apoptosis inhibition and uncontrolled cell proliferation [11-14]. When considering the MSI-H carcinomas of the combined series, *BRAF *exon 15 mutations were detected in 17% of the rectal/sigmoid carcinomas and in 43% of those located elsewhere in the colon. Although no statistically significant association was found with tumor location (*P *= 0.249), *BRAF *mutations were observed more frequently in proximal than in distal carcinomas, which is in agreement with previous studies reporting that *BRAF *mutations occur more frequently in carcinomas arising in the right colon (17.8% versus 3.6% in rectal carcinomas) [13,17]. Several articles found an association between *BRAF *mutations (namely V600E) and the MSI-H phenotype caused by *MLH1 *gene promoter hypermethylation (frequently in the right colon), but the sigmoid and rectal carcinoma in our test and validation series, respectively, with *BRAF *mutation were MSI-H and did not present *MLH1 *promoter hypermethylation [37,38]. On the other hand, when considering the total series of MSI-H carcinomas, *KRAS *mutation frequency was similar for proximal (19%) and distal (25%) carcinomas and inferior to the one observed in microsatellite stable tumors, as previously described [13,14,24]. These findings demonstrate that the MAPK pathway involvement in rectal and sigmoid cancer occurs preferentially by *KRAS *activation.

## Conclusions

Our findings in two independent series from different countries indicate that the pattern of genetic changes involved in rectal and sigmoid carcinogenesis is partially different from that observed elsewhere in the colon. As the mutational spectrum of the MSI pathway appears to differ, the carcinogenic mechanisms may also be distinct between MSI-H carcinomas of the recto-sigmoid and those localized more proximally. Further investigation is warranted to determine the mechanism of MSI-H and its target genes in distal colorectal carcinogenesis both in patients with sporadic or hereditary disease.

## Competing interests

The authors declare that they have no competing interests.

## Authors' contributions

MP, TA, SAD, GEL, IV, CP and VC carried out DNA extractions and genotype analyses and interpretation. MP and TA carried out statistical analysis and drafted the manuscript. OS, MF and and LS contributed with clinical data. LA and CL carried out pathological assessment of the tumors. PL performed and RH interpreted the immunohistochemical experiments. MRT and RAL designed and coordinated the study and contributed to manuscript writing. All authors read and approved the final manuscript.

## Pre-publication history

The pre-publication history for this paper can be accessed here:

http://www.biomedcentral.com/1471-2407/10/587/prepub

## Supplementary Material

Additional file 1 **Clinical and genetic parameters evaluated in the MSI-H tumors according to the large bowel site of origin**. Table presenting the clinical and genetic parameters evaluated in the MSI-H tumors enrolled in this study. Click here for file

Additional file 2 **Somatic mutations detected in *KRAS *exon 2 in rectal and sigmoid cancer patients from the test series**. Table showing the somatic mutations detected in *KRAS *exon 2 in rectal and sigmoid cancer patients from the test series. Click here for file
