# Supplementary material for: Colorectal carcinomas with microsatellite instability display a different pattern of target gene mutations according to large bowel site of origin
Source: BMC Cancer. 2010 Oct 27;10:587. doi: 10.1186/1471-2407-10-587 (PMC2984429; doi:10.1186/1471-2407-10-587)
Supplement: Additional file 1 — Clinical and genetic parameters evaluated in the MSI-H tumors according to the large bowel site of origin. Table presenting the clinical and genetic parameters evaluated in the MSI-H tumors enrolled in this study. [file 1471-2407-10-587-S1.DOC]

**Supplementary table 1 – Clinical and genetic parameters evaluated in the MSI-H tumors according to the large bowel site of origin.**

| **Sample** | **Tumor location** | **Age** | **TNM** | **Gender** | **MSI status** | ***BAX*** | ***IGF2R*** | ***MSH3*** | ***MSH6*** | ***TGFBR2*** | ***MLH1***  **methylation** | ***KRAS*** | ***BRAF*** |
| --- | --- | --- | --- | --- | --- | --- | --- | --- | --- | --- | --- | --- | --- |
| V1 | Caecum | 71 | I | F | MSI-H | MUT | WT | MUT | WT | MUT | n.a. † | WT | n.a. † |
| V2 | Caecum | 65 | II | M | MSI-H | WT | WT | MUT | WT | MUT | n.a. † | WT | n.a. † |
| V3 | Caecum | 61 | III | M | MSI-H | MUT | WT | MUT | WT | MUT | n.a. † | WT | n.a. † |
| V4 | Caecum | 62 | III | F | MSI-H | MUT | WT | MUT | MUT | MUT | UNMET | WT | WT |
| V5 | Caecum | 69 | II | F | MSI-H | WT | WT | MUT | MUT | MUT | n.a. † | WT | n.a. † |
| V6 | Caecum | 74 | I | F | MSI-H | MUT | WT | MUT | WT | MUT | MET | c.184_189del | WT |
| V7 | Caecum | 80 | II | M | MSI-H | WT | WT | MUT | WT | MUT | UNMET | c.49insTGG | WT |
| V8 | Caecum | 75 | III | F | MSI-H | MUT | WT | WT | MUT | MUT | n.a. † | WT | c.1799T>A |
| V9 | ascending colon | 92 | IV | F | MSI-H | WT | WT | MUT | MUT | MUT | UNMET | c.35G>T | WT |
| V10 | ascending colon | 76 | IV | F | MSI-H | WT | WT | MUT | WT | MUT | MET | WT | WT |
| V11 | ascending colon | 69 | III | F | MSI-H | WT | MUT | WT | WT | MUT | MET | WT | c.1799T>A |
| V12 | ascending colon | 89 | II | F | MSI-H | WT | WT | WT | WT | MUT | MET | WT | c.1799T>A |
| V13 | ascending colon | 70 | I | M | MSI-H | MUT | WT | WT | WT | MUT | MET | c.38G>A | WT |
| V14 | ascending colon | 90 | II | F | MSI-H | MUT | MUT | MUT | WT | MUT | MET | WT | c.1799T>A |
| V15 | ascending colon | 84 | II | F | MSI-H | WT | MUT | MUT | WT | MUT | MET | WT | c.1799T>A |
| V16 | ascending colon | 66 | II | F | MSI-H | WT | WT | MUT | WT | MUT | UNMET | WT | WT |
| V17 | right flexure/transversum | 78 | III | M | MSI-H | MUT | WT | WT | WT | WT | UNMET | WT | WT |
| V18 | right flexure/transversum | 71 | II | M | MSI-H | MUT | MUT | MUT | WT | MUT | UNMET | WT | c.1799T>A |
| V19 | right flexure/transversum | 68 | II | F | MSI-H | MUT | MUT | MUT | WT | MUT | MET | WT | c.1799T>A |
| V20 | right flexure/transversum | 79 | IV | F | MSI-H | WT | MUT | WT | WT | MUT | MET | WT | WT |
| V21 | right flexure/transversum | 65 | II | F | MSI-H | MUT | WT | MUT | MUT | MUT | UNMET | WT | WT |
| V22 | right flexure/transversum | 88 | III | F | MSI-H | WT | WT | MUT | WT | MUT | n.a. † | WT | c.1799T>A |
| V23 | left flexure/transversum | 61 | IV | F | MSI-H | WT | MUT | MUT | WT | MUT | UNMET | WT | WT |

**Supplementary table 1 (continued)**

| **Sample** | **Location** | **Age** | **TNM** | **Gender** | **MSI status** | ***BAX*** | ***IGF2R*** | ***MSH3*** | ***MSH6*** | ***TGFBR2*** | ***MLH1* methylation** | ***KRAS*** | ***BRAF*** |
| --- | --- | --- | --- | --- | --- | --- | --- | --- | --- | --- | --- | --- | --- |
| V24 | left flexure/transversum | 66 | II | F | MSI-H | WT | WT | MUT | WT | WT | UNMET | c.38G>A | WT |
| V25 | left flexure/transversum | 67 | II | F | MSI-H | MUT | MUT | MUT | WT | MUT | MET | WT | c.1799T>A |
| V26 | descending colon | 63 | II | F | MSI-H | WT | WT | MUT | WT | WT | n.a. † | WT | n.a. † |
| V27 | sigmoid | 41 | III | F | MSI-H | WT | WT | WT | WT | WT | UNMET | WT | WT |
| V28 | sigmoid | 61 | II | M | MSI-H | WT | MUT | WT | MUT | MUT | UNMET | c.38G>A | WT |
| V29 | sigmoid | 36 | I | M | MSI-H | WT | WT | WT | WT | MUT | n.a. † | WT | n.a. † |
| T27 | sigmoid | 78 | II | M | MSI-H | WT | WT | WT | WT | WT | UNMET | WT | WT |
| T33 | sigmoid | 69 | II | F | MSI-H | WT | WT | WT | WT | WT | UNMET | c.35G>C | WT |
| T35 | sigmoid | 60 | I | F | MSI-H | WT | WT | WT | WT | WT | UNMET | WT | c.1799T>A |
| V30 | rectum | 33 | III | M | MSI-H | WT | WT | WT | WT | MUT | UNMET | WT | WT |
| V31 | rectum | 63 | I | F | MSI-H | WT | MUT | WT | WT | WT | UNMET | WT | c.1799T>A |
| V32 | rectum | 70 | III | M | MSI-H | MUT | WT | WT | WT | MUT | UNMET | c.35G>C/  c.40G>A | WT |
| V33 | rectum | 65 | III | F | MSI-H | WT | WT | WT | WT | WT | n.a. † | WT | n.a. † |
| V34 | rectum | 58 | IV | M | MSI-H | WT | WT | WT | WT | WT | n.a. † | WT | n.a. † |
| V35 | rectum | 75 | III | M | MSI-H | WT | WT | WT | WT | WT | n.a. † | c.38G>A | n.a. † |
| V36 | rectum | 41 | II | M | MSI-H | WT | WT | WT | WT | WT | UNMET | WT | WT |
| T8 | rectum | 59 | III | F | MSI-H | MUT | WT | WT | WT | WT | UNMET | WT | WT |
| T20 | rectum | 74 | II | F | MSI-H | WT | WT | WT | WT | WT | UNMET | WT | WT |
| T41 | rectum | 69 | I | M | MSI-H | MUT | MUT | WT | WT | WT | UNMET | WT | WT |

MSI- microsatellite instability; MSI-H – microsatellite instability-high; WT– wild-type; MUT– mutated; † n.a. – not analyzed; UNMET – unmethylated; MET – methylated; T– test series; V – validation series.
