# Supplementary material for: Colorectal carcinomas with microsatellite instability display a different pattern of target gene mutations according to large bowel site of origin
Source: BMC Cancer. 2010 Oct 27;10:587. doi: 10.1186/1471-2407-10-587 (PMC2984429; doi:10.1186/1471-2407-10-587)
Supplement: Additional file 2 — Somatic mutations detected in KRAS exon 2 in rectal and sigmoid cancer patients from the test series. Table showing the somatic mutations detected in KRAS exon 2 in rectal and sigmoid cancer patients from the test series. [file 1471-2407-10-587-S2.DOC]

**Supplementary table 2** – Somatic mutations detected in *KRAS* exon 2 in rectal and sigmoid cancer patients from the test series.

| **Tumor location** | **Nucleotide change** | **Predicted effect** | **Mutation frequency (%)** |
| --- | --- | --- | --- |
| Rectum  Rectum  Rectum  Rectum  Rectum  Rectum  Sigmoid  Sigmoid  Sigmoid | c.[38G>T; 39C>T]  c.35G>C  c.35G>T  c.38G>A  c.34G>A  c.35G>A  c.38G>A  c.35G>C  c.35G>A | p.Gly13Val  p.Gly12Ala  p.Gly12Val  p.Gly13Asp  p.Gly12Ser  p.Gly12Asp  p.Gly13Asp  p.Gly12Asp  p.Gly12Asp | 1 (8)  1 (8)  1 (8)  3 (23)  3 (23)  4 (30)  1 (20)  1 (20)  3 (60) |
